# *Ginkgo biloba* for Tardive Dyskinesia and Plasma *MnSOD* Activity: Association with *MnSOD* Ala-9Val Variant: A Randomized, Double-blind Trial

**DOI:** 10.2174/1570159X22666240530095721

**Published:** 2024-06-24

**Authors:** Dongmei Wang, Yang Tian, Jiajing Chen, Rongrong Zhu, Jiaxin Li, Huixia Zhou, Dachun Chen, Li Wang, Thomas R. Kosten, Xiang-Yang Zhang

**Affiliations:** 1 CAS Key Laboratory of Mental Health, Institute of Psychology, Chinese Academy of Sciences, Beijing, China;; 2 Department of Psychology, University of Chinese Academy of Sciences, Beijing, China;; 3 Beijing HuiLongGuan Hospital, Beijing, China;; 4 Department of Psychiatry and Behavioral Sciences, Baylor College of Medicine, Houston, Texas, USA

**Keywords:** Schizophrenia, genotype, pharmacogenetics, *ginkgo biloba* extract, tardive dyskinesia, plasma *MnSOD*

## Abstract

**Background:**

Excessive free radicals are implicated in the pathophysiology of tardive dyskinesia (TD), and *Ginkgo biloba* extract (EGb761) scavenges free radicals, thereby enhancing antioxidant enzymes such as mitochondrial manganese superoxide dismutase (*MnSOD*). This study examined whether EGb761 treatment would improve TD symptoms and increase *MnSOD* activity, particularly in TD patients with specific *MnSOD* Val-9Ala genotype.

**Methods:**

An EGb761 (240 mg/day) 12-week double-blind clinical trial with 157 TD patients was randomized. The severity of TD was measured by the Abnormal Involuntary Movement Scale (AIMS) and plasma *MnSOD* activity was assayed before and after 12 weeks of treatment. Further, in an expanded sample, we compared *MnSOD* activity in 159 TD, 227 non-TD and 280 healthy controls, as well as the allele frequencies and genotypes for the *MnSOD* Ala-9Val polymorphism in 352 TD, 486 non-TD and 1150 healthy controls.

**Results:**

EGb761 significantly reduced TD symptoms and increased *MnSOD* activity in TD patients compared to placebo (both *p* < 0.01). Moreover, we found an interaction between genotype and treatment response (*p* < 0.001). Furthermore, in the EGb761 group, patients carrying the Ala allele displayed a significantly lower AIMS total score than patients with the Val/Val genotype. In addition, *MnSOD* activity was significantly lower at baseline in TD patients compared with healthy controls or non-TD patients.

**Conclusion:**

EGb761 treatment enhanced low *MnSOD* activity in TD patients and produced greater improvement in TD symptoms in patients with the Ala allele of the *MnSOD* Ala-9Val polymorphism.

**Clinical Trial Registration No:**

NCT00672373.

## 1 INTRODUCTION

### 1.1 Free Radicals and the Pathophysiological Mechanisms of Tardive Dyskinesia

Long-term antipsychotic medication treatment has an adverse effect known as tardive dyskinesia (TD), which is characterized by repetitive involuntary movements of the perioral area and extremities [1]. Extrapyramidal circuits are involved in regulating the amplitude and velocity of muscle contraction and play an important role in the pathophysiology of TD, but the exact pathophysiological mechanisms of TD have not been elucidated. The neurotoxicity theory of TD hypothesizes that TD may occur due to excessive free radical production by cells during the metabolism of dopamine (DA), which leads to cellular damage [2, 3]. For example, previous studies have shown elevated membrane lipid peroxidation and impaired antioxidant enzyme activity in animal models of TD and in TD patients [4, 5]. Moreover, some antioxidants have been associated with attenuated vacuous chewing movements in rats (a typical animal model of TD) [4], and many antioxidants have been used in the treatment of TD patients, such as vitamin E, vitamin B6, melatonin [6]. However, vitamin B6, and melatonin produce variable clinical results and are currently considered only third-tier drugs [7]. Vitamin E is ineffective and no longer recommended [8]. Taken together, intracellular oxidative stress induced after DA uptake may play a key role in the pathophysiological mechanisms of TD [2, 9]. In addition, dysfunction or degeneration of medium spiny neurons (MSNs) in the extrapyramidal pathway within the striatum has also been implicated in the pathogenesis of TD [10, 11]. Combining these two perspectives, blocking excessive free radicals generated by DA metabolism in the MSNs may play an important role in the treatment and prevention of TD.

### 1.2 Manganese SOD and Its Genetic Polymorphisms in Schizophrenia Patients with TD

Manganese SOD (*MnSOD*) is the main antioxidant defense enzyme in the mitochondria, and its role is to scavenge most of the superoxide anions produced by the electron transport system in mitochondria and defend cells from free radical damage [12, 13]. One possible role of *MnSOD* in the pathogenesis of TD is that antipsychotic drugs increase striatal catecholamine metabolites, leading to excessive production of free radicals, and that *MnSOD* is involved in scavenging excess free radicals in the striatum. Another possible role of *MnSOD* is that it may also play a role in the excitotoxicity of cortical glutamatergic cells. Antipsychotic drugs enhance glutamatergic neurotransmission in the striatum by blocking presynaptic dopamine receptors. There is considerable secondary interaction between glutamatergic and free radical mechanisms, leading to a vicious cycle that promotes oxidative damage in the striatum [14]. Excitotoxicity within glutamatergic neurotransmission may lead to MSNs inactivation of indirect pathway, which is involved in the pathogenesis of movement disorders in Huntington’s disease, Parkinson’s disease and TD [10].

The most widely studied *MnSOD* functional polymorphism in schizophrenia and TD is known as Ala-9Val (rs4880) [15], which is a candidate risk gene associated with superoxide radical detoxification, a putative mechanism for the development of TD. This *MnSOD* gene contains a functional polymorphism Ala-9Val in the mitochondrial targeting sequence (MTS), where the Val allele leads to a conformational change in the MTS that misdirects intracellular transport of the protein [16, 17], and the Ala-Val substitution can alter mitochondrial *MnSOD* activity, potentially inducing free radical accumulation and neuronal damage. The results of previous association studies on *MnSOD* Ala-9Val have been inconsistent, however. A significant association was found between Ala9Val polymorphism and TD by Hori *et al.* (2000) and Galecki *et al.* (2006), for instance [18, 19]. However, these observations have not been replicated by other research groups [20-25]. Additionally, an earlier meta-analysis, which included four studies [18, 20, 21, 23], showed that the *MnSOD*-9Val allele was significantly protective against TD [26]. However, a subsequent meta-analysis [27] including a larger sample size of nine genetic association studies [9, 18, 20-22, 24, 25, 28, 29] did not replicate these positive findings. Therefore, a further investigation of *MnSOD* Ala-9Val polymorphism and TD in schizophrenia is needed.

### 1.3 Pharmacological Mechanisms of Gingko biloba and Its Clinical Application

A German phytopharmaceutical company developed a concentrated, standardized extract of *Gingko biloba* referred to as EGb761 [30]. EGb761 contains 24% flavonoids (including quercetin monosides, biosides and triosides, isorhamnetins, 3’-O-methylmyristicins, and kaempferol), 6% terpenes (including the diterpenes ginkgolide A, B and C as well as the sesquiterpene bilabolide) and less than 5ppm of ginkgolicacides [30-32]. The ingredients of EGb761 have free radical scavenging and antioxidant properties *in vitro* and *in vivo* [30, 31] and are lipid-soluble and can easily cross the blood-brain barrier to provide protection to the brain [30, 31]. Two possible pharmacological mechanisms of EGb761 are (1) direct scavenging of free radicals and (2) indirect inhibition of free radicals by elevating activity of antioxidant enzymes [32]. Reactive oxygen species such as hydroxyl radical (HO•), superoxide anion (O_2_-), hydrogen peroxide (H_2_O_2_), and ferryl ion species can be scavenged by EGb761 [30-32]. It also enhances the activity of antioxidant enzymes such as SOD, glutathione peroxidase (GPx) and catalase (CAT), making it an indirect antioxidant. By scavenging oxyradicals, EGb761 may also lower the generation of free radicals and reduce oxidative stress and the risk of neurotoxicity [30-32]. Clinical studies have shown that long-term administration of EGb761 is useful for those individuals with impaired cognition, mood and related symptoms [33] due to cerebrovascular insufficiency, neurodegeneration or both [34-37]. Our previous study showed that EGb761 increased the efficacy of haloperidol and reduced its extrapyramidal syndrome, possibly due to the antioxidant properties of EGb761 [38, 39]. In another study, we reported that 12 weeks of EGb761 treatment significantly reduced the AIMS total score of TD patients [32]. Based on this published report, the present study explored the role of *MnSOD* and its genetic polymorphism (rs4880) in the improvement of TD by EGb761. Considering the free radical scavenging and antioxidant properties of EGB761 components and the susceptibility of *MnSOD* polymorphism Ala-9Val in TD, we hypothesized that the polymorphism in *MnSOD* Ala-9Val could contribute to EGb761's alteration of *MnSOD* activity in TD patients. This study had the following main aims: (1) whether the *MnSOD* activity would be enhanced in TD patients after 12 weeks of EGb761 treatment; (2) whether the polymorphism Val-9Ala of *MnSOD* would be associated with improved TD and enhanced *MnSOD* activity in TD patients after EGb761 treatment; (3) whether TD patients' *MnSOD* activity would be lower than that of non-TD patients and healthy controls using a subset of expanded samples; and (4) whether the distribution of the Val-9Ala polymorphism of *MnSOD* would differ in TD, non-TD patients and healthy controls and further correlate with *MnSOD* activity.

## 2 METHODS

### 2.1 Subjects

All inpatients were recruited from Beijing Hui-Long-Guan Hospital and HeBei Rong-Jun Psychiatric Hospital between December 2016 and May 2007. Criteria for patient recruitment included: 1) Han Chinese aged 25-75 years; 2) Meeting SCZ diagnostic criteria based on validation of the Chinese version of the validated Structured Clinical Interview for DSM-IV (SCID) by two senior psychiatrists [40]; 3) duration of illness ≥5 years; and 4) taking stable doses of antipsychotic medication for ≥ 6 months prior to study entry.

Also, a group of healthy controls was recruited from the local community. Under the supervision of a research psychiatrist, trained researchers interviewed all healthy controls. They performed a psychiatric evaluation of all healthy controls to rule out those with psychiatric disorders.

All subjects underwent a complete physical examination and reported their medical history. We excluded any subjects with physical illness, alcohol addiction or illicit drug abuse/ dependence. This work complied with the ethical standards of the relevant national and institutional committees on human experimentation and the Helsinki Declaration. An Institutional Review Board at Beijing Hui-Long-Guan Hospital approved the study protocol. Informed consent was obtained from all subjects prior to participation in this study. The trial was registered with ClinicalTrials.gov (NCT00672373).

### 2.2 Clinical Trial of EGb761 in TD Patients

In this clinical trial, a one-week single-blind placebo run-in was followed by a 12-week double-blind treatment period. Patients who improved their AIMS total score by 25% or more after one week of single-blind placebo treatment were eliminated from the trial. 157 patients with TD were randomly assigned to receive either EGb761 capsules or placebo capsules according to the Schooler-Kane research criteria [41] in a double-blind fashion after the run-in period. The capsulized EGb761 was manufactured by Yang Zi Jiang Pharmaceuticals Ltd, Jiangsu, China (batch number 86901749001108). In addition to conventional antipsychotic medication, patients were given either one capsule (80 mg) of EGb761 t.i.d (240 mg/day) or one capsule of the placebo t.i.d for 12 weeks. Antipsychotics and all other medications remained fixed throughout the double-blind period. All study and routine medications were taken by patients in the presence of nursing staff.

### 2.3 Randomization

Patients had a 50/50 chance of being randomized to one of the two groups. Based on a simple randomization list generated by the computer, they were assigned to either the active or placebo groups by an independent third party. This third party employed a protected computer database as the randomization list to ensure that the allocation was hidden. During the double-blind phase, all study personnel and participants were kept unaware of the treatment assignment.

### 2.4 Clinical Measures and Outcomes

We collected detailed information about each subject through a questionnaire. Demographics, detailed medical history, and laboratory tests, including hepatic and renal function, blood routine, and electrocardiogram, were part of the baseline assessment.

We used a validated Chinese translation of the Abnormal Involuntary Movement Scale (AIMS) [42] to assess the severity of TD symptoms based on the total AIMS score (sum of items 1-7). The AIMS is a widely used outcome measure in pharmaceutical trials that is sensitive to changes in TD and has well-defined psychometric features. Patients whose AIMS total score decreased by 30% or more were classified as having a response.

Also, a validated Chinese translation of the Positive and Negative Syndrome Scale (PANSS) was used to assess psychopathology [43]. Results of the AIMS and PANSS as well as laboratory tests were obtained at baseline and at week 12. Prior to the start of the trial, all three psychiatrists who had been in clinical practice for at least five years attended a training session on the use of these scales to ensure consistency and reliability of ratings throughout the study. After the training, their inter-rater correlation values for the AIMS and PANSS total scores were 0.92 and 0.86, respectively.

### 2.5 *MnSOD* Activity and Genotyping of *MnSOD* Ala-9Val Polymorphism

After all, participants fasted the night before, blood samples were collected from all participants' forearm veins between 7 and 9 a.m., and then coded each sample. Plasma samples were then aliquoted and stored at -80^o^C. Without knowing the clinical status of all participants, a research assistant measured *MnSOD* activity according to a previously established procedure in our laboratory [5, 44].

In addition, DNA samples were extracted according to conventional procedures and then stored at -80^o^C. Genotyping of the *MnSOD* Ala-9Val polymorphism was performed according to our previous study [20], using the following primers: sense: 5’-AGCCCAGCCTGCGTAGAC-3’ and antisense: 5’-TACTTCTCCTCGGTGACG-3’. All plasma and DNA samples were used to measure *MnSOD* activity and *MnSOD* Val-9Ala genotype within one year of storage.

### 2.6 Statistical Analysis

This study aimed to assess the effectiveness of EGb761 on TD severity as indicated by AIMS score or clinical symptoms on PANSS, as well as on *MnSOD* activity from baseline to week 12 of treatment by conducting a repeated-measures analysis of variance (ANOVA), with time points (week 0 and week 12) as within-effect and treatment (EGb761 *vs.* placebo) as between-effect. If there was a significant treatment × time interaction, then analysis of covariance (ANCOVA) was used to compare treatment differences in AIMS, PANSS scores or *MnSOD* activity at week 12, using baseline values as covariates. We based power and sample size calculations on a 2-sided significant test at the 0.05 level, with 0.80 power. This calculation yielded a sample size of 128 subjects who would complete 12 week treatment, which was exceeded in this study.

To assess the effect of *MnSOD* Ala-9Val genotype in treatment response on AIMS or PANSS scores, logistic regression models were used to explore the main effect of *MnSOD* Ala-9Val genotype on response rates. After 12 weeks of treatment, we defined patients as responders if their total AIMS total score was reduced by at least 30%. We adopted logistic regression analysis to compare treatment response rates across treatment groups (EGb761 *vs*. placebo) and genotypes (Ala allele carriers *vs.* Val/Val homozygotes).

This study also sought to determine whether improvements in AIMS scores over the course of 12 weeks of treatment were associated with changes in *MnSOD* activity that differed among the *MnSOD* Ala-9Val genotype subgroups. First, we examined Pearson correlations between AIMS improvement and change in *MnSOD* activity in all patients and in each *MnSOD* genotype subgroup, respectively. A stepwise multiple regression analysis was then conducted to confirm the Pearson correlation results.

In the expanded sample, we compared group differences using Χ^2^ test for categorical variables and one-way ANOVA for continuous variables. Next, logistic regression analyses were conducted to explore clinical correlates with TD after other independent variables were controlled for. For *MnSOD* activity, it was analyzed using ANOVA and then ANCOVAs with related variables as covariates. Correlations between variables were performed by Pearson coefficients. Also, we conducted Bonferroni corrections to control for multiple test. The expanded parallel cohort unexposed to EGb761 was included in this study for two purposes. By comparing *MnSOD* activity and *MnSOD* genotype between the healthy control group and the patient group, the first objective was to determine whether *MnSOD* activity had changed and whether the distribution of *MnSOD* genotype in the patient group differed significantly. The second purpose was to investigate whether *Ginkgo biloba* treatment of TD patients resulted in altered and normalized *MnSOD* activity based on the results of the first study, and also to analyze whether the altered *MnSOD* activity caused by *Ginkgo biloba* treatment of TD patients was associated with its improvement of TD symptoms.

We adopted a *χ*^2^ test for goodness of fit to evaluate the degree of deviation from Hardy-Weinberg equilibrium (HWE). Further, we performed a *Χ*^2^ test to compare the genotype and allele distribution of the *MnSOD* Ala-9Val gene polymorphism between TD and non-TD patients and healthy controls. Ala/Ala and Val/Ala genotypes were pooled into a single group for analysis since the frequency of the Ala/Ala genotype was less than 1% in both patients and healthy controls.

## 3 RESULTS

### 3.1 AIMS and PANSS Scores and *MnSOD* Activity During EGb761 Treatment

We reported all the details of this EGb761 clinical trial in a previous study [32]. Repeated-measures ANOVA demonstrated a significant effect of group (F_(1,150)_ = 6.0, *p* < 0.05), time (F_(1,150)_ = 26.2, *p* < 0.001) and group-by-time effects (F_(1,150)_ = 31.9, *p* < 0.001) on the AIMS total score (Table **1**). The AIMS total score in the EGb761 group was substantially lower than in the placebo group at week 12 (F_(1,150)_ = 82.3, *p* <0.001), and remained significant after controlling for demographic and clinical factors (F_(6,144)_ = 56.8, *p* < 0.001). Both the EGb761 and placebo groups improved substantially after 12 weeks of treatment (all *p* < 0.001), without significant differences in PANSS scores between the two groups (all *p* > 0.05).

At baseline, *MnSOD* activity was measured in all 157 TD patients. After 12 weeks of treatment, *MnSOD* activity was available from 66 patients in the EGb761 group and 65 patients in the placebo group. Repeated-measures ANOVA showed a significant group-by-time effect (F_(1,129)_ = 4.03, p < 0.05) and time effect (F_(1,129)_ = 13.9, *p* < 0.001), but a non-significant group effect (F_(1,129)_ = 0.44, *p* = 0.51) (Table **1**). There was a significant difference in *MnSOD* change from baseline to post-treatment between the EGb761 and placebo groups (F_(1, 129)_ = 5.56, *p* < 0.05). Moreover, at week 12, *MnSOD* activity was significantly higher in the EGb group than in the placebo group (F_(1, 129)_ = 6.06, *p* = 0.015). This significant difference in *MnSOD* activity persisted at week 12 after controlling for demographic and clinical variables (F_(8, 121)_ = 6.50, *p* < 0.05).

In the EGb761 group, we did not find any noted correlation between increased *MnSOD* activity and improvement in AIMS score (*p* > 0.05), or improvement in PANSS total and subscale scores (all *p* > 0.05). On the other hand, improvement in the AIMS total score during treatment was linked with baseline *MnSOD* (r = 0.27, df = 1, 66, *p* < 0.05).

### 3.2 Association between *MnSOD* Genotype and Treatment Outcome

As mentioned above, we classified patients as responders if they had at least a 30% reduction in their AIMS total score, and the EGb761 group had more responders than the placebo group (51.3% *vs*. 5.1%; odds ratio 18.4; x^2^=41.6, *p* < 0.0001). Using logistic regression, it was found a significant genotype Χ treatment interaction after 12 weeks of treatment, with an adjusted odds ratio of 1.07 (95% confidence interval 1.04-1.11; x^2^ = 18.55, df = 1, *p* < 0.001; Table **2**). As shown in Fig. (**1**), this interaction showed a higher response rate to EGb761 treatment in the Val/Ala+Ala/Ala group (10/18=55.6% *vs*. 0/15=0%) than in the Val/Val group (30/59=50.8% *vs*. 4/50=8.0%). However, we did not find an effect of genotype on PANSS scores (all *p* > 0.05).

### 3.3 Effect of *MnSOD* Ala-9Val Polymorphism on the Relationship between Improved AIMS and Increased *MnSOD* Activity in EGb761 Treatment

In the EGb761 group, the changes in AIMS score were compared in subgroups by *MnSOD* Ala-9Val genotype. Table **3** exhibits a trend toward a genotype effect (F_(1,71)_ = 3.08, *p* = 0.08), a significant time effect (*p* < 0.001), but no significant genotype × time interaction (*p* = 0.13). At post-treatment, patients carrying the Ala allele (n=17) displayed significantly lower AIMS total score compared with patients with the Val/Val genotype (n=56) (F_(1,71)_ = 7.94, *p* < 0.01) after controlling the variables including age, age of onset, education, BMI, baseline AIMS and PANSS scores.

For *MnSOD* activity, there was a significant time effect (F_(1,67)_ = 8.37, *p* = 0.005), but no significant effect of *MnSOD* genotype (F_(1,67)_ = 1.41, *p* = 0.24) and no genotype × time interaction (F_(1,67)_ = 0.09, *p* = 0.76). After treatment, we found no noticeable difference between patients carrying Val/Val genotype (n = 53) and those carrying the Ala allele (n=16) in their *MnSOD* activity (F_(1,67)_ = 2.03, *p* = 0.16).

In addition, in the EGb761 group, no significant association was observed between improved AIMS score and increased *MnSOD* activity was observed in all patients, or in two patient subgroups by Ala-9Val genotype (all *p* > 0.05).

### 3.4 Demographic and Clinical Variables, *MnSOD* Plasma Activity and Ala-9Val Gene Polymorphism in TD, non-TD and Healthy Control Groups at Baseline

In an expanded sample, we further recruited 352 TD and 486 non-TD patients, as well as 1150 healthy controls. The average duration of illness for all patients was 24.6 ± 9.4 years, and the average number of hospitalizations was 4.2 ± 2.8. The average dose of antipsychotic medication (chlorpromazine equivalent) was 415 ± 221 mg/day.

We compared the genotype and allele frequencies of Ala-9Val of *MnSOD* in all TD, non-TD patients, and healthy controls (Table **4**). From these subjects, we then randomly selected 159 TD, 227 non-TD patients and 280 healthy controls to measure their *MnSOD* activity. Demographic and clinical variables between the two cohorts (*MnSOD* genotyping and activity testing) were not significantly different (all *p* > 0.05). The distribution of *MnSOD* Ala-9Val genotypes in TD, non-TD patients and healthy controls conformed to HWE (all *p* < 0.05). As shown in Table **4**, the frequencies of *MnSOD* Ala-9Val genotypes and alleles were not significantly different between SCZ patients with and without TD and healthy controls, nor were they significantly different between TD and non-TD patients (all *p* > 0.05). Further, TD patients had significantly lower *MnSOD* activity than non-TD patients (17.1 ± 9.6 U/ml *vs*. 19.8±11.8 U/ml, F_(1, 384)_ = 4.66, *p* < 0.05). When clinical variables were controlled for, this significant difference remained (F_(6, 372)_ = 4.53, *p* < 0.05). Additionally, compared with healthy controls (22.0 ± 12.3U/ml), both TD and non-TD patients had significantly lower *MnSOD* activity (F_(2,663)_ = 8.57, *p* < 0.0001) (TD *vs*. controls: *p* < 0.001; non-TD *vs*. controls: *p* < 0.05). After adjusting for demographic and clinical variables, both differences remained significant (both *p* < 0.05). Furthermore, plasma *MnSOD* activity did not differ significantly between TD and non-TD patients grouped by typical or atypical antipsychotic treatment (all *p* > 0.05).

## 4 DISCUSSION

### 4.1 Low *MnSOD* Activity in TD Patients

At baseline, plasma *MnSOD* activity was lower in TD patients than in non-TD patients and healthy controls, which is consistent with previous reports showing lower SOD in plasmas, red cell and CSF of TD patients [6, 14]. *MnSOD* is a pivotal enzyme in the antioxidant defense system that scavenges most of superoxide anions generated by the mitochondrial electron transport system [12]. *MnSOD* is also associated with the development of the nervous system and is of particular implications for the termination of neural growth and the initiation of differentiation [12]. Therefore, the low *MnSOD* activity supports the neuronal degeneration hypothesis of TD [2, 45].

### 4.2 Improved Dyskinetic Symptoms and Increased *MnSOD* Activity in TD Patients Treated with EGb761

Importantly, we found that EGb761 treatment markedly improved dyskinetic symptoms and increased *MnSOD* activity in TD patients. Generally, TD has developed due to long-term exposure to antipsychotic medications, which, by blocking dopamine receptors, may lead to increased dopamine turnover and metabolism, resulting in increased production of free radicals [45]. Excessive free radicals may attack neuronal cell membrane, resulting in membrane and functional alterations, and subsequent neurotoxicity/neurodegeneration, which are involved in the pathophysiological mechanisms of TD [14]. On the other hand, many pre-clinical and clinical studies have shown that EGb761 possesses potent antioxidant and free radical-scavenging activities, and has protective effects against free-radical-induced cellular damage and dysfunction [46]. Our findings provide further evidence that EGb761 treatment increased *MnSOD* activity, suggesting that EGb761 treatment may directly enhance antioxidant enzyme activity in TD patients. However, in this study, the increase in *MnSOD* activity was not associated with an improvement in AIMS score in patients receiving EGb761, which may be due to limited sample size or due to confounding factors that may interfere with this association. Another factor may lie in the use of the AIMS tool to measure TD symptoms. The complex distinction between spontaneous and activated movements in AIMS makes it difficult to score movements reliably [47], which may explain why AIMS 1-7 scores did not correlate with *MnSOD* activity in this study. In addition, some consensus has emerged regarding the optimal use of AIMS [48], which remains the gold standard for the clinical assessment of TD, with its imperfections. According to the TD assessment workshop, clinical trials should implement a method that tolerates inter-rater reliability (*e.g*. blinded central video rater consensus). Furthermore, multifaceted methods of analysis, such as effect size, minimal clinically important differences, response analysis, and category shifts, may provide broader evidence of clinical validity [48].

### 4.3 Association of *MnSOD* Ala-9Val Genotype with Response to EGb761 Treatment in TD Patients

**Fig. (2):**
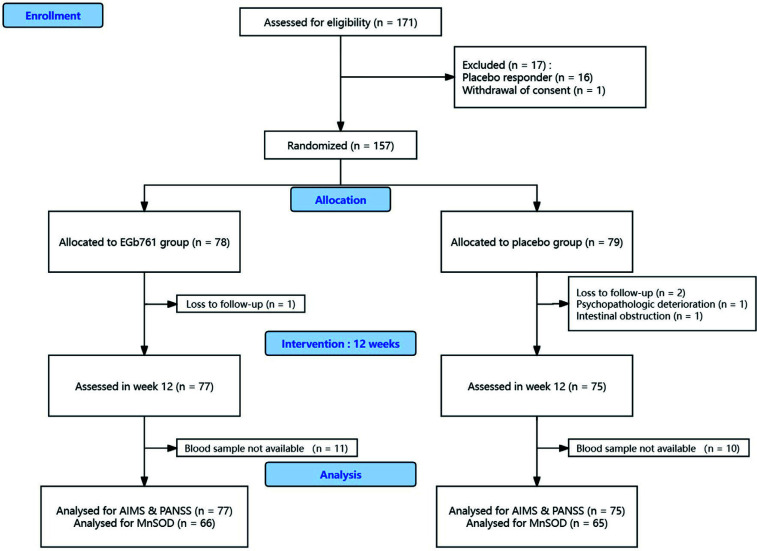
CONSORT flow diagram for trial EGb-761 and Placebo for Tardive Dyskinesia [32].

Interestingly, we further observed that the *MnSOD* Ala-9Val genotype was associated with EGb761 treatment response in TD patients, and that patients with the Ala allele responded better to EGb761 treatment than patients with the Val/Val genotype (Fig. **2**). It is important to elucidate the mechanisms underlying the pharmacogenetic effects of EGb761 in the treatment of TD. The neurobiological interpretation of our findings may be correlated with the effect of the *MnSOD* Ala-9Val genotype on *MnSOD* activity. The *MnSOD* gene has a functional polymorphism called Ala-9Val, whose variant from T to C causes the amino acid Val to convert to Ala. This alteration results in the target domain's chromosomal structure to shift from a beta-sheet to an alpha-helix [16, 49]. The effectiveness of enzyme transport to the mitochondria matrix is improved by the alpha-helical structure with the Ala precursor in comparison to the beta-sheet structure [50]. Thus, *MnSOD* Val-9Ala may play a particularly important role in the treatment of TD by EGb761 by affecting mitochondrial *MnSOD* concentrations Since patients with the Ala allele showed better improvement with EGb761 treatment in our current study, we speculate that these patients with the Ala allele may have greater *MnSOD* activity, which contributes to more free radical scavenging and stronger antioxidant effects of EGb761. It is then worth mentioning that although the *MnSOD* Ala-9Val genotype affects the helix structure of the *MnSOD* protein in mitochondria, its relationship with plasma *MnSOD* activity is unclear and needs to be further explored. In addition, plasma *MnSOD* activity at baseline was positively associated with a reduction in AIMS score. This implies patients with greater baseline *MnSOD* activity responded better to EGb761 treatment, which was correlated with the *MnSOD* Ala allele, providing indirect evidence that patients with higher *MnSOD* activity or with the Ala allele may response better to EGb761 treatment. However, we did not observe a direct association between *MnSOD* activity and Ala-9Val genotype in both patients and healthy controls. Therefore, the origin of peripheral *MnSOD*, the mechanisms by which EGb761 treatment affects *MnSOD* activity, and the correlation between *MnSOD* Ala-9Val genotype and the response to EGb761 treatment in TD patients will warrant further investigation. EGb761 may also act through antioxidant-independent mechanisms. Previous studies have shown that EGb761 can protect against Aβ-induced neurotoxicity by reducing tau hyperphosphorylation and thus treat AD [51]. Furthermore, EGb761 inhibits tau phosphorylation at Ser262 in primary cortical neurons of rats through three pathways: (1) ROS inhibition directly within cells; (2) direct inhibition of GSK3β phosphorylation, and (3) inhibition of GSK3β through downregulation of ROS [52]. Although it is unclear from this study whether EGb761 may alter GSK3β phosphorylation in TD patients, in-depth studies should be conducted to explore possible antioxidant-independent mechanisms of this pathway in the action of EGb761.

### 4.4 Limitations

There are several limitations in this study. First, a major limitation of this type of study is that the composition of EGb761, a phytotherapeutic agent, is not uniform. When standardizing an extract, the levels of one (or a few) of the components may be adjusted, but the levels of other components may vary considerably and therefore may produce different pharmacological effects. As a result, the clinical effects can vary considerably from batch to batch. Second, only *MnSOD* activity was measured without other antioxidant parameters. The sequential and cooperative activities of complex antioxidant defense systems produce effective antioxidant effects, such as numerous antioxidant enzymes and non-enzymatic antioxidant molecules. Therefore, measurement of one antioxidant enzyme activity can only partially reveal free radical-mediated neuronal dysfunction. Third, since the *MnSOD* polymorphism Val-9Ala directly affects the expression of *MnSOD* rather than *MnSOD* activity, *MnSOD* expression should be measured simultaneously in future studies to examine whether similar conclusions can be drawn. Fourth, it remains uncertain whether peripheral *MnSOD* activity is associated with *MnSOD* activity in the brain. There is no direct evidence that peripheral *MnSOD* may reflect similar changes in *MnSOD* activity in the brain. Therefore, the relationship between peripheral and brain *MnSOD* activity warrants further investigation. Finally, although Loonen *et al.* developed a new tool called the Schedule of Instruments for Assessing Drug-induced Dyskinesia (SADIMoD) for all relevant movement disorders [47, 53, 54], it is important to note that the AIMS remains the gold standard for clinical assessment of TD, although it is not perfect. It is very important to use AIMS properly under optimal conditions. There are ways to optimize data collection, inter-rater reliability, and use of the AIMS [48]. In this study, instead of reporting means of crude TD scores, alternative analyses of AIMS data, such as mean minus score changes at each time point, should also be conducted in further studies. Such changes in subtraction scores could better determine whether improvements in the scale are relevant, for example, the minimal clinically important difference (MCID) was estimated as a 2-point reduction on items 1-7 of the AIMS [55].

## CONCLUSION

In conclusion, our study demonstrated that 12 weeks of EGb761 treatment improved TD symptoms and enhanced low *MnSOD* activity, especially in TD patients with the Ala allele of the *MnSOD* Ala-9Val polymorphism. We also found that baseline plasma *MnSOD* activity was positively corrected with reduction in AIMS score. These findings suggest that patients with higher baseline *MnSOD* activity responded better to EGb761 treatment, which correlates with the *MnSOD* Ala allele, indirectly demonstrating that patients with higher *MnSOD* activity or with the Ala allele may respond better to EGb761 treatment.

## Figures and Tables

**Fig. (1):**
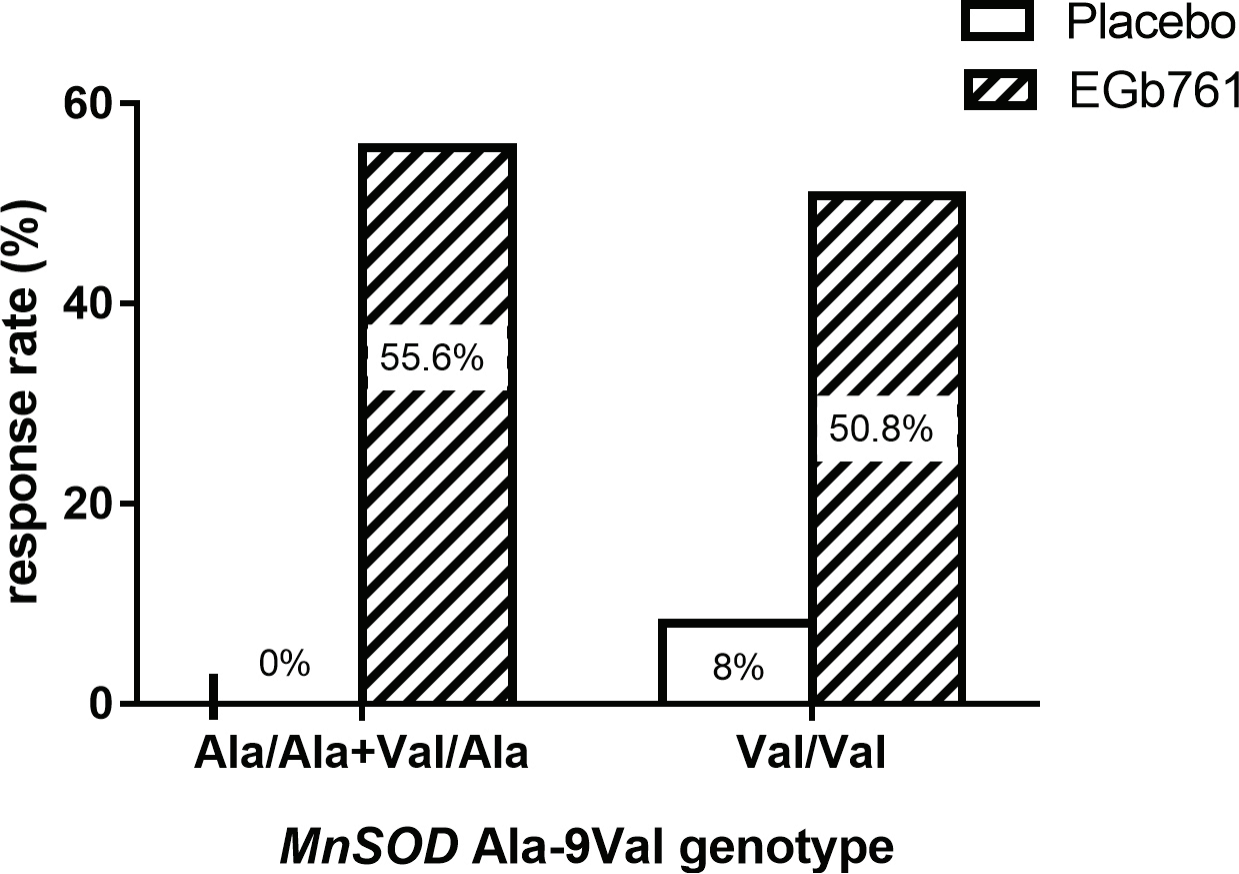
Response rates by *MnSOD* Ala-9Val genotype after 12 weeks of EGb761 treatment. There was a significant interaction between Ala-9Val genotype and treatment (adjusted OR = 1.07, 95% confidence interval 1.04 -1.11, *p* < 0.001).

**Table 1:** AIMS score and *MnSOD* activity at baseline and week 12 in EGb and placebo groups.

**-**	**Baseline**	**Week 12**	**Group F (*p* Value)**	**Time F (*p* Value)**	**Group×Time F (*p* Value)**
AIMS total score	-	-	6.0 (0.02)	26.2 (<0.001)	31.9 (<0.001)
EGb	7.0 ± 2.9	4.9 ± 2.2*	-	-	-
Placebo	6.9 ± 3.6	7.0 ± 3.3	-	-	-
EGb-responder	7.7 ± 3.3	4.3 ± 2.3	-	-	-
*MnSOD* activity	-	-	0.44 (0.51)	13.9 (<0.001)	4.03 (< 0.05)
EGb	16.1 ± 9.6	21.5 ± 6.9	-	-	-
Placebo	17.2 ± 8.7	19.0 ± 6.2	-	-	-
EGb-responder	17.3 ± 7.0	22.9 ± 7.8	-	-	-

**Note:** # A total of 157 TD patients participated in this 12-week clinical trial. After treatment, 5 patients dropped out including 1 from EGb761 group and 4 from placebo group.

*indicates the comparison between EGb761 and placebo groups at post-treatment. *p < 0.05.

**Table 2:** Logistic regression models of treatment response at 12-week.

**Prediator**	**OR (95% CI)**	**Χ2**	***p* Value**
Age	1.05 (0.99-1.13)	2.44	0.12
Education	1.06 (0.88-1.28)	0.35	0.56
*MnSOD* genotype	1.01(0.918-1.10)	0.01	0.93
EGb Treatment	16.49 (5.46-49.85)	24.67	< 0.001
*MnSOD* genotype*Treatment	1.07 (1.04-1.11)	18.55	< 0.001

**Abbreviations: ***MnSOD* = manganese Superoxide dismutase; CI = confidence interval; OR = odds ratio.

**Table 3:** Longitudinal changes in AIMS score and plasma *MnSOD* activity in tardive dyskinesia patients treated with EGb761, grouped by *MnSOD* Ala-9Val genotypes.

**Parameter**	**Val/Val**	**Val/Ala+ Ala/Ala**	**Genotype F (*p* Value)**	**Time F (*p* Value)**	**Genotype×Time F (*p* Value)**
AIMS total score	-	-	3.08 (0.08)	81.8 (<0.001)	2.4 (0.13)
Baseline	7.3(3.1)	6.6 (2.6)	-	-	-
Post-treatment	5.1(2.1)	3.6(1.7) **	-	-	-
*MnSOD* activity	-	-	1.41 (0.24)	8.37 (< 0.01)	0.09 (0.76)
Baseline	17.9(8.6)	15.4 (8.7) *	-	-	-
Post-treatment	20.0(6.1)	18.0(8.9)	-	-	-

**Note:** *indicates the comparison between genotypes at post-treatment. *p < 0.05, **p < 0.01.

**Table 4:** Demographics, clinical parameters, *MnSOD* allele and genotype distribution of TD and non-TD patients and healthy controls.

**Parameter**	**TD** **(n = 352)**	**Non-TD** **(n = 486)**	**Controls** **(n = 1150)**	**Χ^2^ or F**	***p* Value**
Sex (male/female)	324/28	379/107***	774/376	91.0	< 0.001
Age (years)	49.3(9.2)	47.3(9.9) ***	43.6±12.8	56.63	< 0.001
Education (years)	8.5(2.6)	9.4(6.4) ***	9.5±5.6	2.46	0.09
Current Smoker (%)	242(68.8%)	292(60.1%)	594(60.1%)	8.99	< 0.05
Age of onset (years)	23.7(5.2)	23.4(5.8)	-	0.11	0.71
Hospitalization Times	4.6(3.1)	4.0(2.7) ***	-	9.92	< 0.01
Antipsychotic dose(mg/day)	441.2(325.7)	482.2(435.3)	-	2.03	0.19
PANSS Total score	61.4(14.6)	59.5(15.1)	-	2.75	0.06
P subscore	11.3(4.5)	12.0(5.4)	-	2.21	0.14
N subscore	24.8(7.8)	21.8(8.4)	-	24.46	<0.001
G subscore	25.3(5.9)	25.6(6.1)	-	0.61	0.48
*MnSOD* Ala-9Val polymorophism	-	-	-	-	-
Allele frequency (%)	-	-	-	-	-
Val	606(86.1%)	835(85.9%)	2031(88.3%)	4.75	0.09
Ala	98(13.9%)	137(14.1%)	269(11.7%)	-	-
Genotype frequency (%)	-	-	-	9.43	0.051
Val/Val	258(73.3%)	350(72.0%)	889(77.3%)	-	-
Ala/Val	90(25.6%)	135(27.8%)	253(22%)	-	-
Ala/Ala	4(1.1%)	1(0.2%)	8(0.7%)	-	-

**Note:** TD = tardive dyskinesia, PANSS = Positive and Negative Syndrome Scale, P = positive symptom, N = negative symptom, G = General psychopathology.

*indicates the comparison between TD and non-TD patients. *p < 0.05, **p < 0.01, ***p < 0.001.

## Data Availability

The data that support the findings of this study are available from the corresponding author, [X.Y.Z.], upon reasonable request.

## LIST OF ABBREVIATIONS

CAT: Catalase
GPx: Glutathione Peroxidase
MCID: Minimal Clinically Important Difference
MSNs: Medium Spiny Neurons
MTS: Mitochondrial Targeting Sequence
TD: Tardive Dyskinesia

## STANDARDS OF REPORTING

Consort guidelines and methodology were followed.
